# Medication adherence among persons with coronary heart disease and associations with blood pressure and low-density-lipoprotein-cholesterol

**DOI:** 10.1007/s00228-022-03276-4

**Published:** 2022-01-21

**Authors:** Elisabeth Pedersen, Raul Primicerio, Kjell H. Halvorsen, Anne Elise Eggen, Beate Hennie Garcia, Henrik Schirmer, Marit Waaseth

**Affiliations:** 1 Department of Pharmacy, UiT The Arctic University of Norway, Tromsø, Norway; 2 Faculty of Biosciences, Fisheries and Economics, UiT, The Arctic University of Norway, Tromsø, Norway; 3 Department of Community Medicine, UiT The Arctic University of Norway, Tromsø, Norway; 4 Department of Cardiology, Akershus University Hospital, Lørenskog, Norway; 5 Institute of Clinical Medicine Campus Ahus, University of Oslo, Oslo, Norway

**Keywords:** Medication adherence, Coronary heart disease, Lipid-lowering drugs, Antihypertensive drugs, Acetylsalicylic acid

## Abstract

**Purpose:**

To describe medication adherence to lipid-lowering drugs (LLDs), antihypertensive drugs, and acetylsalicylic acid (ASA) among persons with coronary heart disease (CHD) and explore its association with low-density-lipoprotein (LDL)-cholesterol, and systolic and diastolic blood pressure.

**Methods:**

Based on record linkage between the seventh wave of the Tromsø Study and the Norwegian Prescription Database, medication adherence was calculated as the proportion of days covered (PDC) for persistent prevalent users in the period of 365 days before the attendance date. Multivariable linear regression models were used to assess the association between systolic and diastolic blood pressure and medication nonadherence to antihypertensive drugs, age, sex, lifestyle, body mass index (BMI), current and previous diabetes, and between LDL-cholesterol and medication nonadherence to LLDs, age, sex, lifestyle, BMI, and current and previous diabetes.

**Results:**

Mean PDC was 0.94 for LLDs and antihypertensive drugs and 0.97 for ASA. Among persons with PDC ≥ 0.80 for LLDs, 12.0% had an LDL-cholesterol < 1.8 mmol/L. Blood pressure < 140/90 mmHg (< 140/80 mmHg if diabetes patient) was reached by 55.1% of those with a PDC ≥ 0.80 for antihypertensive drugs. Adherence to LLDs was associated with lower LDL-cholesterol, while neither systolic nor diastolic blood pressure was associated with adherence to antihypertensive drugs.

**Conclusion:**

Adherence to antihypertensive drugs, LLDs, and ASA among persons with CHD were high despite low achievement of treatment goals for blood pressure and LDL-cholesterol. There was a statistically significant association between adherence to LLDs and LDL-cholesterol, but not between adherence to antihypertensive drugs and blood pressure.

**Supplementary Information:**

The online version contains supplementary material available at 10.1007/s00228-022-03276-4.

## Introduction

Adherence to medications for secondary prevention of coronary heart disease (CHD) is important to achieve the full effect of lipid-lowering drugs (LLDs), antihypertensive drugs, and low-dose acetylsalicylic acid (ASA) and thereby avoid new cardiovascular events [1–4].

Lowering low-density-lipoprotein (LDL)-cholesterol and blood pressure reduce the risk of further morbidity and mortality of coronary heart disease [5, 6]. European guidelines for prevention of cardiovascular disease have recommended that patients with established CHD should have a blood pressure of < 140/90 mmHg (< 140/80 mmHg in patients with diabetes) and an LDL-cholesterol of < 1.8 mmol/l (< 70 mg/dL) [7, 8]. In the more recent guidelines concerning management of chronic coronary syndromes and those concerning hypercholesterolemia, the recommendations for persons with a very high risk of new coronary events are now further reduced to an LDL-cholesterol reduction of ≥ 50% from baseline and an LDL-cholesterol goal of < 1.4 mmol/L (< 55 mg/dL) [9, 10]. Risk factor control among persons with CHD is found to be suboptimal, also in population-based studies [11–14]. Treatment goal achievement for LDL-cholesterol is particularly low. Suboptimal medication adherence is a possible explanation for the poor treatment goal achievement. Several studies have found an association between being adherent and achieving LDL-cholesterol or blood pressure control [15–19].

Adherence to long-term therapies is generally found to be as low as 50% [20]. Although some studies have found slightly higher adherence to medications used for secondary prevention of CHD, there is still potential for improvement [1, 17, 21]. Proportion of days covered (PDC) and medication possession ratio (MPR) are two common measures used to assess adherence. Being adherent is often defined as having a PDC or MPR of ≥ 80% [20]. Although this cut-off is considered arbitrary, some studies have found that a medication adherence of ≥ 80% for medications used for cardiovascular diseases is associated with fewer adverse coronary events [4, 22].

The medication adherence process can be divided into three separate phases: initiation, implementation, and discontinuation. Initiation determines when the first dose is taken, implementation indicates to which extent patients’ actual dose per medication corresponds to the prescribed regimen and is often measured as a proportion, while discontinuation marks the last dose taken and thus the end of treatment [23]. Persistence is defined as the time between initiation and discontinuation.

Few studies have assessed the association between medication adherence and LDL-cholesterol and systolic and diastolic blood pressure as continuous measures. Adherence to LLDs, antihypertensive drugs, and ASA among persistent prevalent medication users with CHD has also not been properly described.

This study aims to describe medication adherence to LLDs, antihypertensive drugs, and ASA among persons with CHD, focusing on the implementation phase of the adherence process, and explore its association with LDL-cholesterol serum concentrations, and systolic and diastolic blood pressure.

## Methods

### Data sources

The data for this study were retrieved from the seventh wave of the Tromsø Study (Tromsø 7, conducted in 2015–2016) and the Norwegian Prescription Database (NorPD).

The Tromsø Study is a Norwegian population-based epidemiological health study that has been conducted seven times since 1974. The population of the Tromsø Study consists of inhabitants in the municipality of Tromsø in North Norway, a university town with approximately 73,000 inhabitants in 2016. Tromsø 7 invited all inhabitants in the municipality aged 40 years or older (*n* = 32,591). Attendance rate was 65% (*n* = 21,083).

Data collection includes questionnaires, interviews, biological sampling, and clinical examinations from where we extracted blood pressure and anthropometric measurements (height and weight), LDL-cholesterol values, and self-reported diseases, lifestyle (smoking, alcohol consumption, diet, and physical activity), and demographic information.

Data from Tromsø 7 were linked with NorPD data using the unique national identity number assigned to all citizens in Norway. NorPD contains information on all prescriptions dispensed to individuals from Norwegian pharmacies. Medications given at hospitals, nursing homes, or over-the-counter are not included. We extracted the following variables: date of dispensing and information on medications dispensed, including Anatomical Therapeutic Chemical (ATC) code [24] and the number of dosage units dispensed. Prescribed daily dosage is not available in NorPD, and we therefore assumed a daily dosage of one dosage unit (e.g. tablet or capsule).

### Study population

The study population consisted of participants reporting established CHD (*n* = 1483), defined as previous myocardial infarction, present or previous angina pectoris, previous percutaneous coronary intervention, or coronary artery bypass graft surgery.

### Medications included

**Fig. 1:**
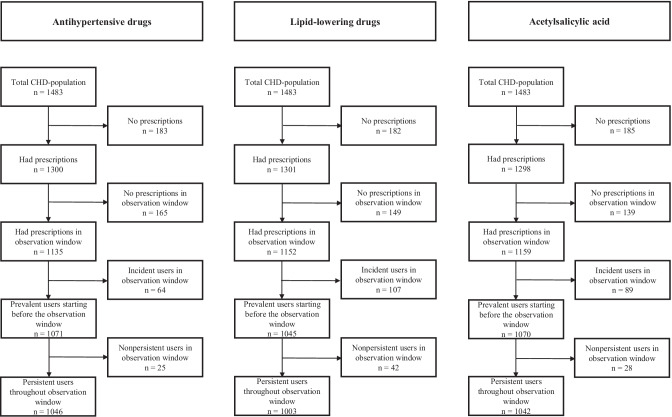
Flowchart of medication users

From NorPD, we included use of medications for secondary prevention of CHD based on the prevailing European clinical guidelines in 2015/2016. This included ASA, LLDs (mainly statins), and antihypertensive drugs (angiotensin-converting enzyme (ACE) inhibitors, angiotensin receptor blockers (ARBs), beta-blockers, calcium channel blockers (CCBs), thiazides, and other antihypertensives) [7]. ATC codes for the medications included can be found in Online Resource 1. The number of participants using the different medication groups and subgroups can be found in Fig. 1 and Online Resource 2.

### Adherence measurement

**Fig. 2:**
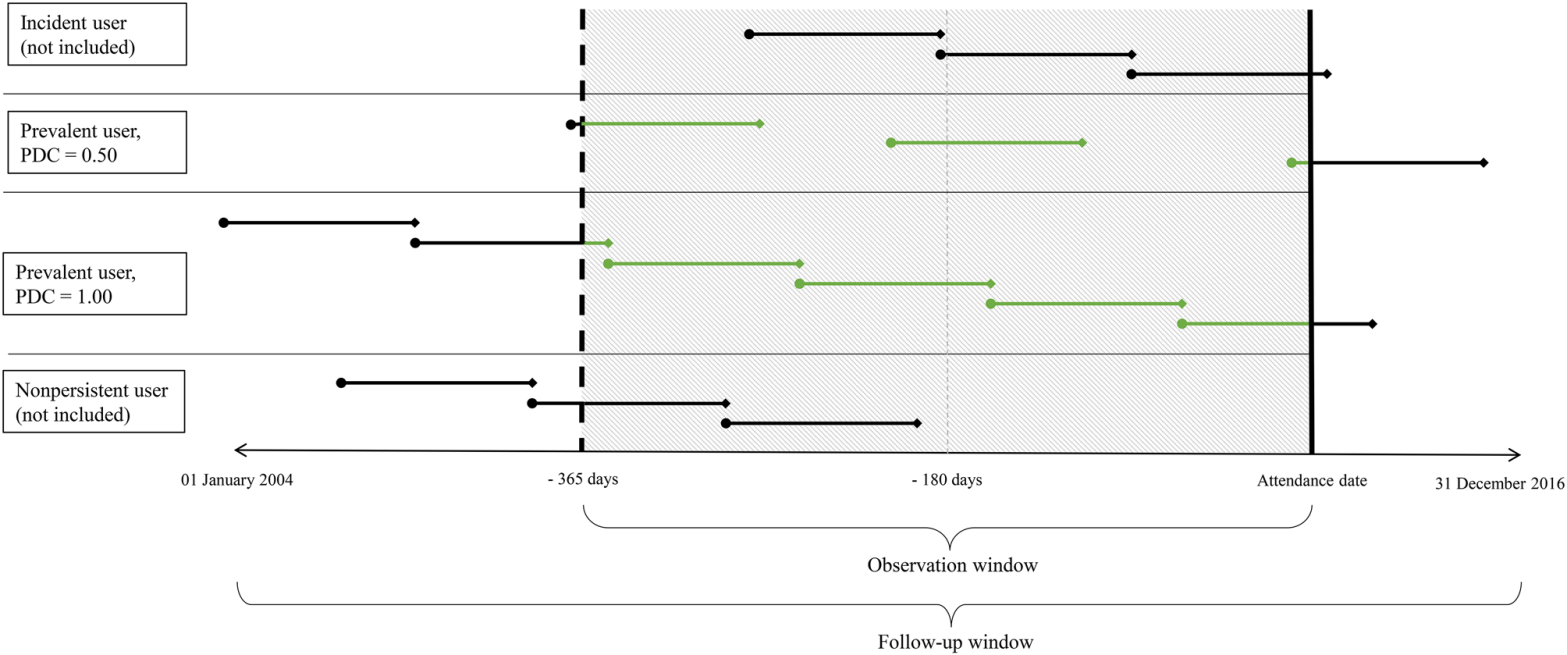
Defining proportion of days covered for persistent prevalent medication users. Treatment period durations were defined by the number of medication units (e.g. tablets) dispensed at each treatment fill (dots). PDC was calculated for persistent prevalent users based on treatment durations during the observation window (green in digital version, grey in print). The mid panel shows one participant with 50% adherence and one with perfect adherence according to PDC. Incident users (top) and nonpersistent users (bottom) were excluded. PDC, proportion of days covered

We calculated adherence to medication use as PDC, calculated as a continuous multiple-interval measure of medication availability (CMA) 7 in the R-package AdhereR [25]. CMA7 is defined as “number of gap days for all event intervals extracted from the total time interval; (accounting for carrying over from before the observation window and within the observation window, and excluding the supply left at the observation window end)” [25, 26]. The observation window was set from 365 days before the attendance date in Tromsø 7 until the attendance date (see Fig. 2). The follow-up window was set from the 1st of January 2004 until the 31st of December 2016 to use all available data from NorPD. The medications supplied before the beginning of the observation window could then be carried over into the observation window if the days supplied extended into this period. Medication supplied from prescriptions filled before the end of the previous supplies was also carried forward to after the end of the previous days supplied. This was only done within the same 5th level ATC-code (chemical substance level) to avoid overestimating medication supplies in connection to switches of medications within the same medication group.

Adherence calculations were done for persistent prevalent users, defined as participants who had used the medications from before the start of the observation window and had supplies available to cover days within 180 days before attendance in Tromsø 7. Incident and nonpersistent users were excluded (see Figs. 1 and 2). Incident users were defined as participants who had not filled any prescriptions for the relevant medications within 365 days before the first prescription in the observation window. These were excluded because they had too few dispensing of the relevant medications before attending Tromsø 7 for the calculated PDC to be reliable. Adherence estimations during short time intervals are found to be imprecise, and it is therefore recommended to calculate PDC only when the observation window is long enough to last at least three dispensings or over 9 months [26]. In Norway, a typical dispensing of LLDs, antihypertensive drugs, or ASA lasts about 3 months; hence, four dispensings should cover 1 year. Nonpersistent users were defined as those not having any days covered with the relevant medications within 180 days before the attendance date. These were excluded from our analyses because discontinuation is a different step in the adherence process, and those who discontinue treatment could therefore be different than those who have poor implementation [23]. In a previous study, we have also considered those without medications dispensed within 180 days before attending Tromsø 7 as not being medication users at the time of attendance [27]. Our focus in the current study was thereby on the implementation phase within the adherence taxonomy [23].

For users of several antihypertensive drugs and LLDs, a day was considered covered when at least one of the medications was available [19, 28]. See Fig. 1 for an overview of medication users per medication group; for subgroups, see Online Resource 2.

### Measurement of LDL-cholesterol and blood pressure

In Tromsø 7, blood pressure was measured by trained personnel using a digital automated device (Dinamap ProCare 300 monitor, GE Healthcare, Norway). Three measurements were taken with 1-minute intervals and after 2 minutes of seated rest [12]. In the analyses, we used the mean of the two final measurements, except if the third measurement was missing (*n* = 2), then we only used the second measurement. If both the second and third measurements were missing (*n* = 1), we used the first measurement. Three participants did not have any blood pressure measurements registered and were excluded from the analyses examining blood pressure. Achieving the treatment goal for blood pressure was defined as having a blood pressure < 140/90 mmHg (< 140/80 mmHg in those with diabetes) [7].

LDL-cholesterol was collected and analysed by trained personnel using enzymatic colorimetric methods with commercial kits on a Cobas 8000 c702 (Roche Diagnostics GmbH, Mannheim, Germany) from non-fasting venous blood samples. The analysis was performed at the Department of Laboratory Medicine, University Hospital of North Norway, Tromsø, Norway (ISO certification NS-EN ISO 15189:2012) [12]. Eleven participants did not have any LDL-cholesterol measurements registered and were excluded from the analysis of LDL-cholesterol. The treatment goal for LDL-cholesterol was set to < 1.8 mmol/L based on the European guidelines from 2012 which were the prevailing guidelines at the time of Tromsø 7 [7].

### Covariates

Weight and height were measured with light clothing and no shoes to the nearest 0.1 kilogram and 0.1 centimetre using the Jenix DS-102 height and weight scale (DongSahn Jenix, Seoul, Korea). We calculated body mass index (BMI) as weight in kilograms divided by height in metres squared.

We collected variables concerning current or previous diabetes and lifestyle from questionnaires in Tromsø 7. Having a diagnosis of diabetes was defined as answering “yes, currently” or “previously, not now” when asked “Have you ever had, or do you have diabetes?” (answering options “no”, “yes, currently” and “previously, not now”) or reporting current use of antidiabetic drugs, either by reporting a brand name of an antidiabetic drug when asked to state the name of all medicines used regularly during the last 4 weeks, or checking off “now” when asked “Do you use or have you used tablets for diabetes/insulin?”. Participants were considered not having diabetes if they did not reply that they had diabetes and did not report using any antidiabetic drug.

Two variables summarizing lifestyle were obtained using multidimensional scaling, computed with the R-package vegan, applied to a multivariate dataset including variables concerning self-reported smoking, alcohol use, diet, and physical activity. For more information about these variables, see Online Resource 3.

### Statistical analysis

Descriptive statistics are presented as proportions and means with standard deviation (SD). We applied three multivariable linear regression models to assess the association between systolic and diastolic blood pressure and medication nonadherence to antihypertensive drugs, age, sex, lifestyle, BMI, current and previous diabetes (models i and ii), and between LDL-cholesterol and medication nonadherence to LLDs, age, sex, lifestyle, BMI, and current and previous diabetes (model iii). Medication nonadherence was assessed as the adherence variables had to be reversed and log-transformed (1.1—log(PDC)) in these analyses (skewness in variables). The analyses were done as complete case analyses, hence excluding participants with missing values in the relevant variables. The significance level was set to 5%.

The analyses were conducted using R (R Core Team (2021), R: A language and environment for statistical computing. R Foundation for Statistical Computing, Vienna, Austria. URL https://www.R-project.org/).

### Ethics

The study was approved by the Regional Committee for Medical and Health Research Ethics of North Norway (2015/1775) and had an approved Data Protection Impact Assessment (DPIA) from UiT The Arctic University of Norway. All participants in the Tromsø Study have given written informed consent for their data to be used in research.

## Results

Participants defined as persistent prevalent medication users were 1003 for LLDs, 1046 for antihypertensive drugs, and 1042 for ASA (Fig. 1). The number of participants that had been dispensed prescriptions for all three medication groups was 701, while 113 participants had not had any dispensed prescriptions for any of three. Characteristics of the total study population and users of each of the medication groups are shown in Table 1.

**Table 1:** Characteristics of the study population and the different subgroups

		**Study population** ***n* = 1483**		**Users of** **lipid-lowering drugs** ***n* = 1003**		**Users of antihypertensive drugs** ***n* = 1046**		**Users of acetylsalicylic acid** ***n* = 1042**	
Age (years), mean (SD)		68.7	(10.8)	69.5	(9.6)	70.7	(9.8)	69.7	(9.7)
Sex, *n* (%)									
Male		1037	(69.9)	730	(72.8)	730	(69.8)	765	(73.4)
BMI (kg/m^2^), mean (SD)		28.4	(4.5)	28.4	(4.3)	28.7	(4.5)	28.4	(4.3)
Diabetes, *n* (%)									
Current		204	(11.1)	160	(16.0)	172	(16.4)	158	(15.2)
Previous		21	(1.4)	14	(1.4)	15	(1.4)	11	(1.1)
LDL-cholesterol (mmol/L), mean (SD)		2.9	(1.0)	2.6	(0.8)	2.7	(0.9)	2.7	(0.9)
Systolic blood pressure (mmHg), mean (SD)		135.9	(20.9)	136.5	(20.7)	137.1	(21.1)	136.6	(20.4)
Diastolic blood pressure (mmHg), mean (SD)		74.4	(9.9)	74.3	(9.7)	73.8	(9.7)	74.2	(9.8)

*BMI* body mass index, *LDL* low-density-lipoprotein, *SD* standard deviation

**Fig. 3:**
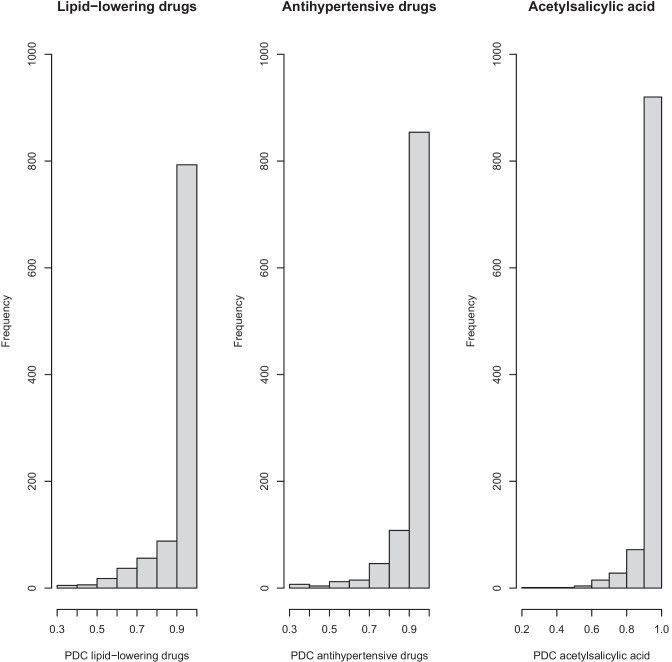
Distributions of proportion of days covered. PDC, proportion of days covered

Medication adherence was high, with a mean PDC of ≥ 0.94 for all medication groups and subgroups (Table 2). The distribution of PDC in all medication groups is shown in Fig. 3.

**Table 2:** Adherence to antihypertensive drugs, lipid-lowering drugs, acetylsalicylic acid, and subgroups

	**PDC, mean (SD)**		**Proportion of participants with** **PDC ≥ 0.80, *n* (%)**	
Antihypertensive drugs (*n* = 1046)	0.94	(0.10)	963	(92.1)
ACE-inhibitor (*n* = 215)	0.98	(0.07)	208	(96.7)
ARB (*n* = 371)	0.96	(0.10)	348	(93.8)
Beta-blocker (*n* = 759)	0.96	(0.10)	708	(93.3)
CCB (*n* = 269)	0.97	(0.08)	259	(96.3)
Thiazide (*n* = 229)	0.95	(0.12)	205	(89.5)
Lipid-lowering drugs (*n* = 1003)	0.94	(0.12)	884	(88.1)
Statin (*n* = 987)	0.94	(0.12)	869	(88.0)
Acetylsalicylic acid (*n* = 1042)	0.97	(0.08)	992	(95.2)

*ACE* angiotensin-converting enzyme, *ARB* angiotensin receptor blocker, *CCB* calcium channel blocker, *PDC* proportion of days covered, *SD* standard deviation

Treatment goals for both systolic and diastolic blood pressure (< 140/90 mmHg, < 140/80 mmHg if diabetic) were reached for 54.7% of the antihypertensive drug users. The treatment goal for systolic blood pressure was reached by 56.8%, while 90.9% reached the goal for diastolic blood pressure. The proportion of participants reaching the blood pressure goal among participants with a PDC ≥ 0.80 (*n* = 963) was 55.1% compared to 49.4% among those with PDC < 0.80 (*n* = 83).

For the LLD-users, the proportion reaching the treatment goal for LDL-cholesterol (< 1.8 mmol/L or < 70 mg/dL) was 11.2%. The proportion of participants reaching the treatment goal among participants with a PDC ≥ 0.80 (*n* = 884) was 12.0% compared to 5.0% among those with a PDC < 0.80 (*n* = 119).

The regression models (Table 3) show that an increased systolic blood pressure was significantly associated with higher age (*β* = 0.31, *p* < 0.001), while an increased diastolic blood pressure was significantly associated with lower age (*β* = *− *0.14, *p* < 0.001), male sex (*β* = 0.09, *p* = 0.009), and lifestyle (*β* = 0.10, *p* = 0.008). None of the blood pressure measurements were significantly associated with adherence to antihypertensive drug use. An increase in LDL cholesterol was significantly associated with nonadherence to LLDs (*β* = 0.12, *p* < 0.001), female sex (*β* = − 0.12, *p* < 0.001), lifestyle (*β* = 0.14, *p* < 0.001), and not having current diabetes (*β* = − 0.09, *p* = 0.009).

**Table 3:** Multivariable linear regression models (i, iii, and iii) showing factors associated with systolic and diastolic blood pressure and LDL-cholesterol

*Predictors*	**Systolic blood pressure (i)**				**Diastolic blood pressure (ii)**				**LDL-cholesterol (iii)**			
	*B*	*SE B*	*β*	*p*	*B*	*SE B*	*β*	*p*	*B*	*SE B*	*β*	*p*
Constant	87.990	8.300	0.000	< .001	81.698	3.882	0.000	< .001	2.807	0.303	0.000	< .001
Nonadherence antihypertensive drugs^1^	1.647	1.484	0.035	.267	0.876	0.694	0.040	.207				
Nonadherence lipid-lowering drugs^2^									0.190	0.050	0.124	** < .001**
Age	0.683	0.077	0.314	** < .001**	− 0.141	0.036	− 0.141	** < .001**	0.001	0.003	0.014	.714
Sex, male	− 2.282	1.551	− 0.048	.142	1.901	0.726	0.088	**.009**	− 0.203	0.061	− 0.115	** < .001**
Lifestyle 1^3^	− 4.471	8.481	− 0.019	.598	10.600	3.967	0.099	**.008**	1.174	0.322	0.138	** < .001**
Lifestyle 2^3^	− 7.705	8.580	− 0.028	.369	− 2.277	4.013	− 0.018	.571	− 0.209	0.328	− 0.021	.523
BMI	0.208	0.155	0.043	.180	0.098	0.072	0.045	.175	0.008	0.006	0.044	.191
Diabetes, current	− 0.785	1.837	− 0.014	.669	− 1.027	0.859	− 0.038	.232	− 0.189	0.072	− 0.087	**.009**
Diabetes, previous	− 2.246	5.445	− 0.013	.680	0.089	2.547	0.001	.972	− 0.017	0.212	− 0.003	.934
Observations	939				939				909			
*R*^2^/*R*^2^-adjusted	0.107/0.099				0.071/0.063				0.050/0.042			

*B* unstandardized beta,* β* standardized beta, *BMI* body mass index, *LDL* low-density-lipoprotein, *PDC* proportion of days covered, *SE* standard error

^1^1.1-log(PDC antihypertensive drugs)

^2^1.1-log(PDC lipid-lowering drugs)

^3^Lifestyle variables constructed using multidimensional scaling including self-reported smoking, alcohol use, diet, and physical activity. Higher values of lifestyle 1 indicate an unhealthier lifestyle with more consumption of alcohol and red meat as well as more current smokers. Higher values of lifestyle 2 indicate more frequent consumption of omega 3

The regression models indicated that the predictors explained 9.9% of the variance in systolic blood pressure (adjusted *R*^2^ = 0.099, *F*(8,930) = 13.91, *p* < 0.001), 6.3% of the variance in diastolic blood pressure (adjusted *R*^2^ = 0.063, *F*(8,930) = 8.84, *p* < 0.001), and 4.2% of the variance in LDL-cholesterol (adjusted *R*^2^ = 0.042, *F*(8,900) = 5.96, *p* < 0.001).

## Discussion

In this study, we have identified a high medication adherence, defined as proportion of days covered, to antihypertensive drugs, lipid-lowering drugs, and acetylsalicylic acid among persons with CHD. Despite the high adherence, achievement of treatment goals for blood pressure and LDL-cholesterol was low. From the regression models, we found that adherence to LLDs was significantly associated with a lower LDL-cholesterol, but no significant association was identified between adherence to antihypertensive drugs and lower blood pressure. Sex and lifestyle were associated with both LDL-cholesterol and diastolic blood pressure, while age was associated with both systolic and diastolic blood pressure.

Previous studies examining medication adherence to secondary prevention of CHD or cardiovascular disease using pharmacy dispensing data have also found high adherence for LLDs with mean PDC of 0.76 [29] or 79.8% having PDC ≥ 0.80 [17]. Also, adherence to antihypertensive drugs is found to be high with mean PDC of 0.77 [15]. The medication adherence found in the present study is even higher than what has been seen in these studies. There could be several explanations for this. First, we have only selected persistent prevalent medication users to enable calculation of PDC for the whole year before attendance in Tromsø 7. Most previous studies have included new users in the first months or years after treatment initiation or included a combination of new and prevalent users. The highest discontinuation rates have been found to appear in the first year after treatment initiation, and persistent users tend to have higher adherence than those who are nonpersistent [30]. Second, disease severity has been associated with higher adherence, and thus, we anticipate higher adherence to secondary prevention of CHD compared with primary prevention [29]. Third, NorPD covers all dispensed medications in these medication groups, irrespective of reimbursement, and none of these medications is available over-the-counter in Norway. This enables us to include all the medications that are actually available to the participants, which may not be the case in all other studies. Altogether, these patients seem to be highly adherent.

Despite the low nonadherence to LLDs in this patient group, it was significantly associated with a higher LDL-cholesterol. This agrees with other studies showing that adherence to LLDs is associated with reaching the recommended treatment goals for LDL-cholesterol [17, 18]. A Norwegian study by Munkhaugen et al. also found that self-reported medication adherence to statins was associated with both lower LDL-cholesterol and achievement of the treatment goal for LDL-cholesterol [31]. Lowering LDL-cholesterol reduces the risk of a new coronary event [5]. In a previous study, we showed that only 9% of these participants reached the treatment goal of < 1.8 mmol/L (< 70 mg/dL) [11], which is surprisingly seen in the light of the high adherence shown in the current study. Even if more focus on the importance of adherence could lead to an increase in treatment goal achievement in this patient population, other actions such as increasing the prescribed daily dose of statins or adding ezetimibe might also be necessary. In the current study, we were not able to identify how much the LDL-cholesterol had been reduced from baseline, or whether dose increase could be justified. Previous studies have shown a larger LDL-cholesterol reduction with more intense statin treatment [5, 32, 33], and a more intense treatment seems to be necessary in our population. However, it is important to keep in mind that higher dosages of statins are more prone to give side effects, which again might negatively affect the participants’ adherence.

Neither systolic nor diastolic blood pressure was significantly affected by adherence to antihypertensive drugs in our analyses. This contrasts with other studies, where being adherent was associated with achieving blood pressure goals [15, 16, 19]. However, a Norwegian study by Sverre et al. also found no association between adherence to antihypertensive drugs, based on self-report, and blood pressure control [34]. In the same study, increased blood pressure was found to be associated with older age and higher BMI. We have previously found that 42% of our study population did not reach the recommended blood pressure goal (140/90 mmHg or 140/80 mmHg if they also had diabetes) and that self-reported use of antihypertensive drugs was not associated with achieving the treatment goal [11]. When we now have identified such a high adherence to these drugs among the persistent prevalent medication users in the same population, but no association with their blood pressure, a potential explanation could be that treatment intensity is too low. Our results also show the very clear association between higher age and higher blood pressure, which could be caused by arterial stiffening which increases with age and is associated with higher blood pressure [35]. It is also possible that elderly persons are treated less intensely with antihypertensive drugs than younger persons, which might be clinically sound. This has also been taken into account by the more recent European clinical guidelines from 2016 [8], contrary to the guidelines from 2012 [7] applied in this study. Treating hypertension in elderly patients can be challenging, as they are usually more frail and more sensitive to potentially harmful side-effects such as reduced kidney function and orthostatic hypotension, which could lead to falls [36]. Although reducing blood pressure is very important in CHD patients, it might not be possible, or even appropriate, to bring all patients to the recommended blood pressure goal.

Though we have not assessed initiation and persistence in this study, Fig. 1 shows that about 12% of the participants had no prescriptions dispensed for each of these medication groups throughout the whole follow-up window from 2004 until they attended Tromsø 7, indicating that they either never had such medications prescribed, or that they never initiated treatment. Of those who had been dispensed prescriptions for either of the medication groups, 15% discontinued antihypertensive treatment or LLDs and 13% discontinued ASA before attending Tromsø 7. The proportion of participants discontinuing treatment is lower than what has been found in previous Nordic studies [30, 37, 38], indicating that our study population does have good persistence. However, those who discontinue or do not initiate treatment might have an even higher risk of new coronary events. It should be further investigated how to identify these patient groups and assessed whether a closer treatment follow-up is warranted.

### Strengths and limitations

A strength of this study was the use of two reliable data sources; the Tromsø Study, a reliable population-based data source with high attendance rate, where measurements of blood pressure and cholesterol were performed by trained personnel using standardized procedures and instruments, and NorPD, which includes information about all dispensings from Norwegian pharmacies. Using a follow-up window from 2004, when NorPD was established, enabled us to capture as many days covered with medication supplies as possible in the observation window, and hence estimate how much medication the participants had available during the observation window. Furthermore, the medications studied herein are prescription-only medications; we should therefore have captured all medications available to the participants. However, as we did not have any information about potential hospital stays, and medications dispensed to patients in hospitals are not included in NorPD, this could potentially lead to a slight underestimation of PDC.

One limitation is that we did not have information about prescribed daily dose as this is not available in NorPD. We therefore assumed a daily dose of one dosage unit. As most medications used for secondary prevention of coronary heart disease are taken once daily this is a fair assumption, though some medications, particularly some antihypertensives, might have a higher daily dosage (units per day), and this would lead us to overestimate the PDC. However, a validation of the self-reported use of these medications in this population showed that a daily dose of one dosage unit a day was a more accurate assumption than one defined daily dose (DDD) a day, which would have been the alternative [27].

As in other studies evaluating refill adherence, we cannot determine that having had the medications dispensed actually means that they have been consumed by the participants. We can however be quite certain that these medications are considered in use by the participants, as previously shown in the validation study [27].

When measuring PDC for combination therapies for either antihypertensive or lipid-lowering treatment, we considered a day to be covered if the participants had at least one medication available. We could therefore not determine if the participants using combination therapies used all the antihypertensive drugs or LLDs prescribed, and this might have led us to overestimate the true adherence.

Finally, and perhaps most importantly, the proportions of variance in LDL-cholesterol, and in systolic and diastolic blood pressure, explained by our models were low (4.2–9.9%), indicating that other factors also contribute to the observed variance. As this is an observational study, our results might also have been influenced by unmeasured confounders.

## Conclusion

Adherence to lipid-lowering drugs, antihypertensive drugs, and acetylsalicylic acid among persons with coronary heart disease was high despite low achievement of treatment goals for blood pressure and LDL-cholesterol. Adherence to lipid-lowering drugs was significantly associated with lower LDL-cholesterol, while adherence to antihypertensive drugs was not significantly associated with either systolic or diastolic blood pressure. This suggests that these participants might not receive optimal medication treatment and that perhaps dosages or numbers or combinations of medications are insufficient. More research is needed to explore this.

## Supplementary Information

Below is the link to the electronic supplementary material.

Supplementary file1 (PDF 12 KB)

Supplementary file2 (PDF 39 KB)

Supplementary file3 (PDF 203 KB)

## Data Availability

The data that support the findings of this study are available from the Tromsø Study and the Norwegian Institute of Public Health, but restrictions apply to the availability of these data, which were used under licence for the current study, and so are not publicly available. Data are however available from the authors upon reasonable request and with permission of the Tromsø Study, the Norwegian Institute of Public Health, and the Regional Committees for Medical and Health Research Ethics.
