# Supplementary material for: Medication adherence among persons with coronary heart disease and associations with blood pressure and low-density-lipoprotein-cholesterol
Source: Eur J Clin Pharmacol. 2022 Jan 21;78(5):857–67. doi: 10.1007/s00228-022-03276-4 (PMC9005431; doi:10.1007/s00228-022-03276-4)
Supplement: Supplementary file 1 — Supplementary file1 (PDF 12 KB) [file 228_2022_3276_MOESM1_ESM.pdf]

Supplementary table 1: Overview of ATC-codes included in the three medication categories

Medication	ATC-codes
Acetylsalicylic acid	
Acetylsalicylic acid	B01AC06
Lipid lowering drugs	
Statins	C10AA, C10BA
Other lipid lowering drugs	C10AC, C10AX, C10
Antihypertensive drugs	
ACE inhibitors	C09A, C09B
ARBs	C09C, C09D
Beta-blockers	C07
CCBs	C08, C09BB, C09DB, C09DX01, C09DX03
Thiazides	C03A, C03EA, C07B, C09BA, C09DA, C09DX01, C09DX03
Other antihypertensives	C02, C03C, C03D, C03EA, C03X

Abbreviations: ACE, angiotensin converting enzyme; ARB, angiotensin receptor blocker; ATC, anatomical therapeutic chemical; CCB, calcium channel blocker
