# Supplementary material for: Medication adherence among persons with coronary heart disease and associations with blood pressure and low-density-lipoprotein-cholesterol
Source: Eur J Clin Pharmacol. 2022 Jan 21;78(5):857–67. doi: 10.1007/s00228-022-03276-4 (PMC9005431; doi:10.1007/s00228-022-03276-4)
Supplement: Supplementary file 2 — Supplementary file2 (PDF 39 KB) [file 228_2022_3276_MOESM2_ESM.pdf]

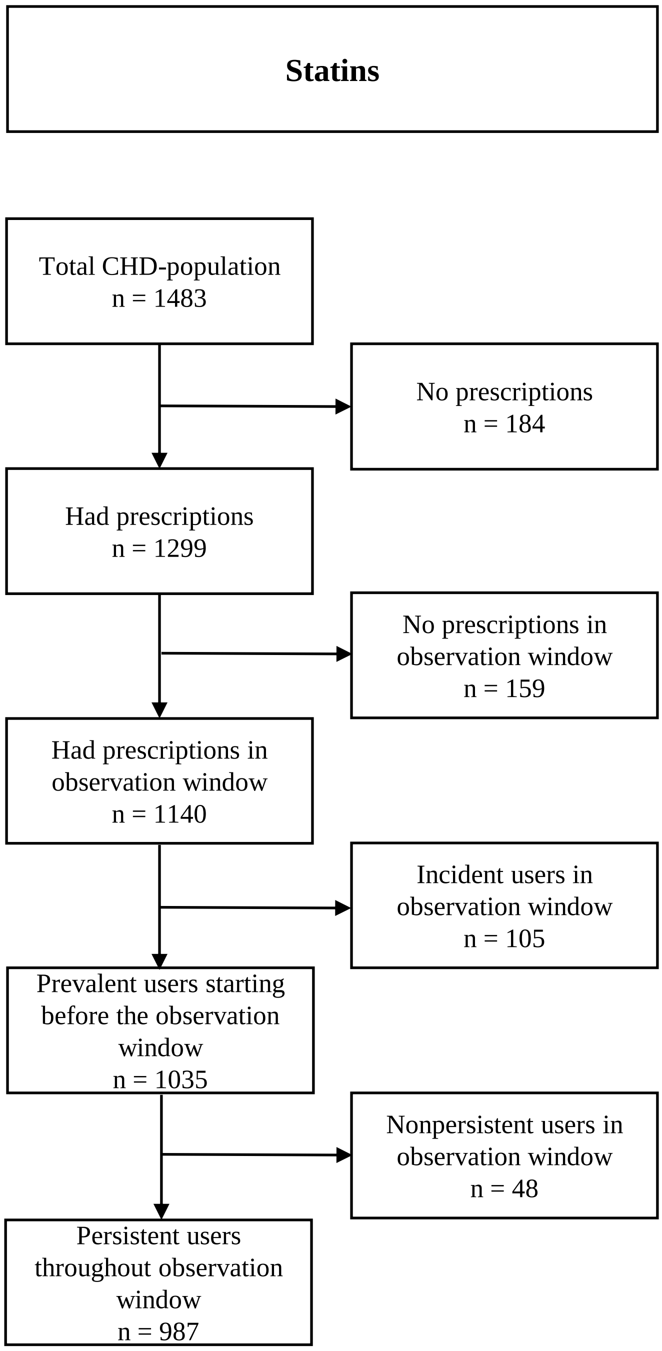
Statins
Total CHD-population n = 1483
No prescriptions n = 184
Had prescriptions n = 1299
No prescriptions in observation window n = 159
Had prescriptions in observation window n = 1140
Incident users in observation window n = 105
Prevalent users starting before the observation window n = 1035
Nonpersistent users in observation window n = 48
Persistent users throughout observation window n = 987

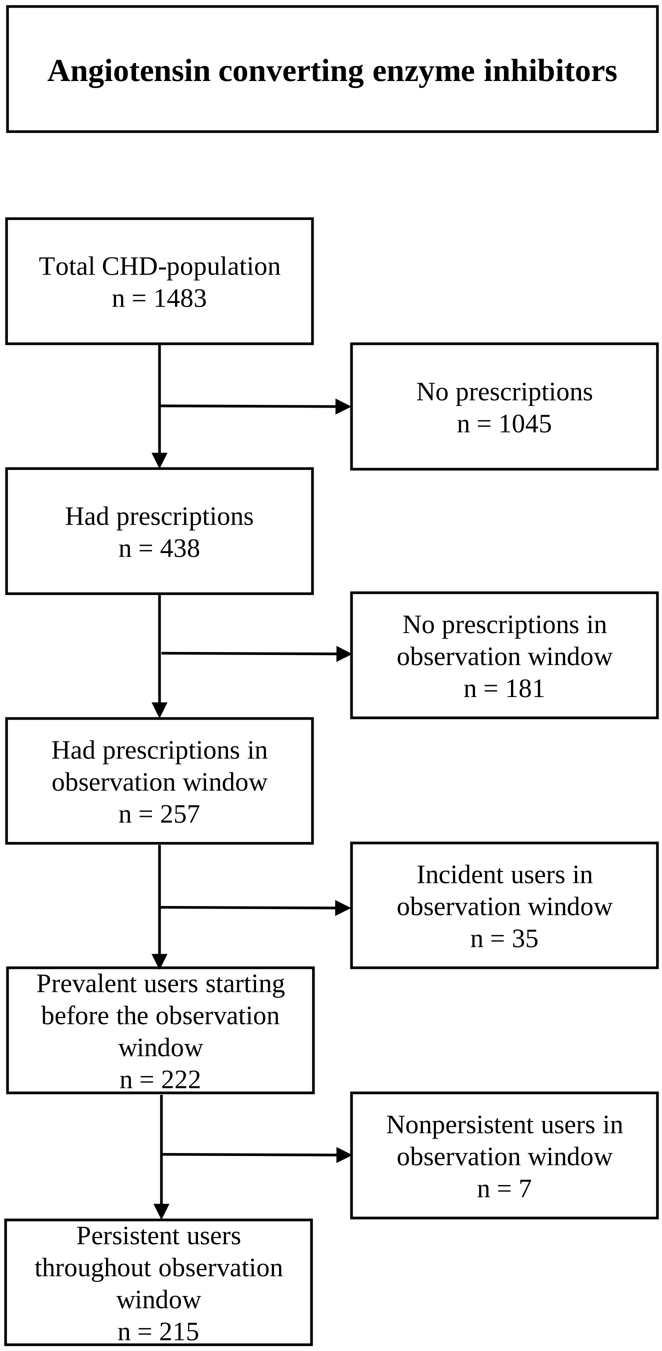
Angiotensin converting enzyme inhibitors
Total CHD-population
n = 1483
No prescriptions
n = 1045
Had prescriptions
n = 438
No prescriptions in observation window
n = 181
Had prescriptions in observation window
n = 257
Incident users in observation window
n = 35
Prevalent users starting before the observation window
n = 222
Nonpersistent users in observation window
n = 7
Persistent users throughout observation window
n = 215

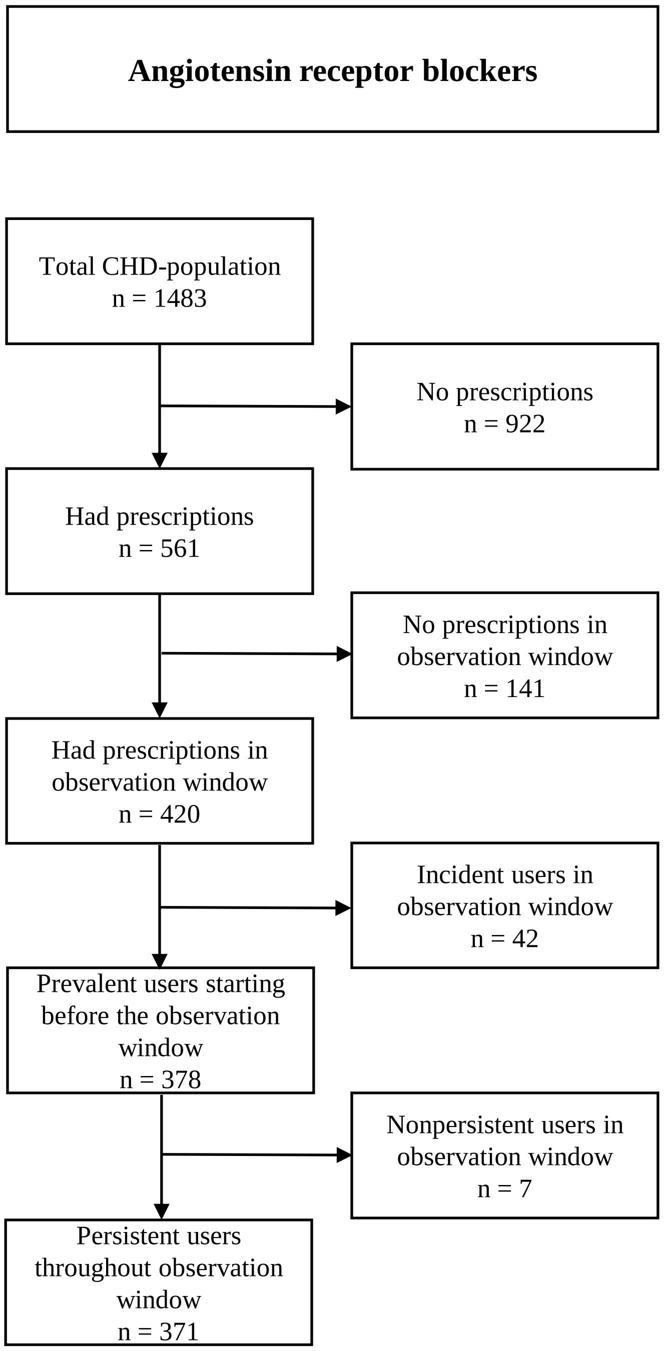
Angiotensin receptor blockers
Total CHD-population n = 1483
No prescriptions n = 922
Had prescriptions n = 561
No prescriptions in observation window n = 141
Had prescriptions in observation window n = 420
Incident users in observation window n = 42
Prevalent users starting before the observation window n = 378
Nonpersistent users in observation window n = 7
Persistent users throughout observation window n = 371

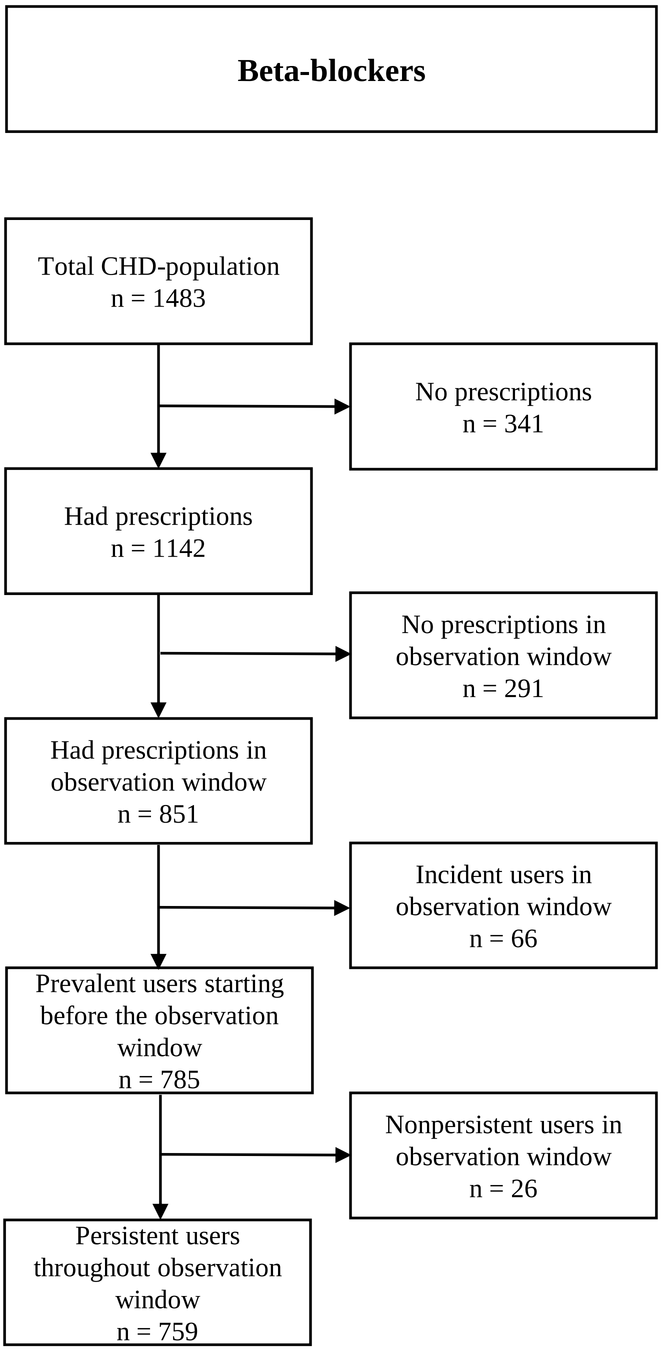
Beta-blockers
Total CHD-population n = 1483
No prescriptions n = 341
Had prescriptions n = 1142
No prescriptions in observation window n = 291
Had prescriptions in observation window n = 851
Incident users in observation window n = 66
Prevalent users starting before the observation window n = 785
Nonpersistent users in observation window n = 26
Persistent users throughout observation window n = 759

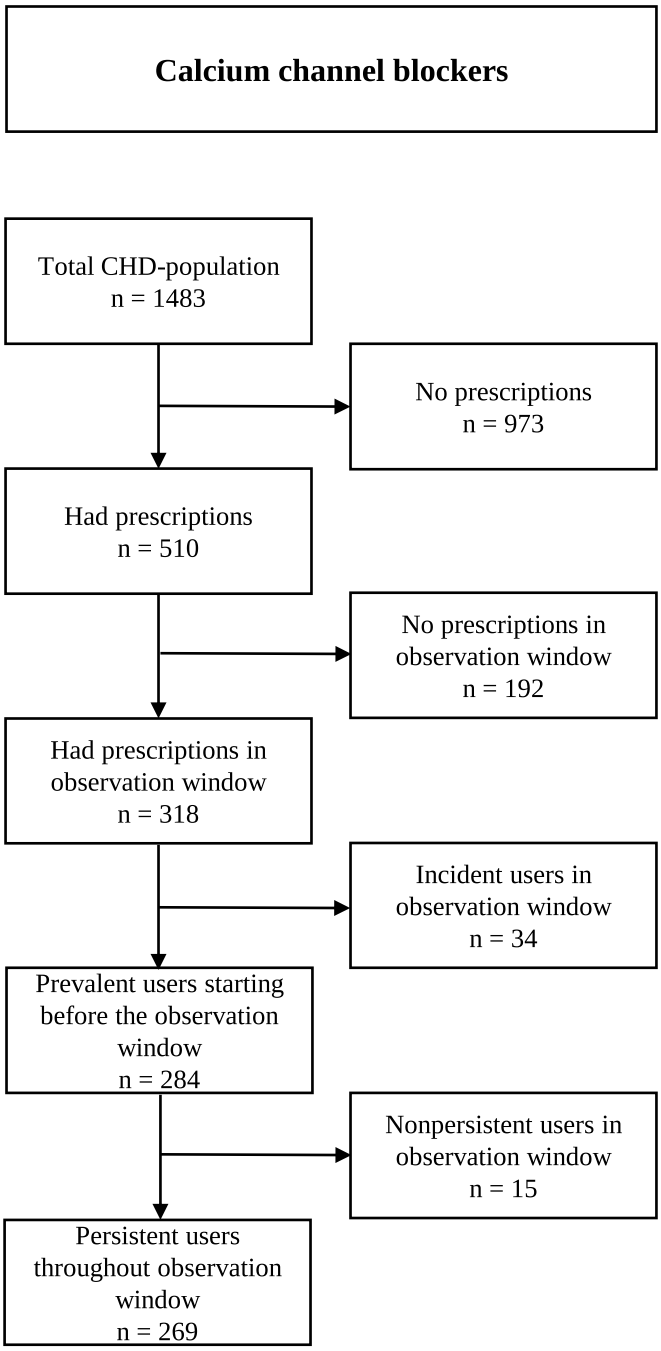
Calcium channel blockers
Total CHD-population n = 1483
No prescriptions n = 973
Had prescriptions n = 510
No prescriptions in observation window n = 192
Had prescriptions in observation window n = 318
Incident users in observation window n = 34
Prevalent users starting before the observation window n = 284
Nonpersistent users in observation window n = 15
Persistent users throughout observation window n = 269

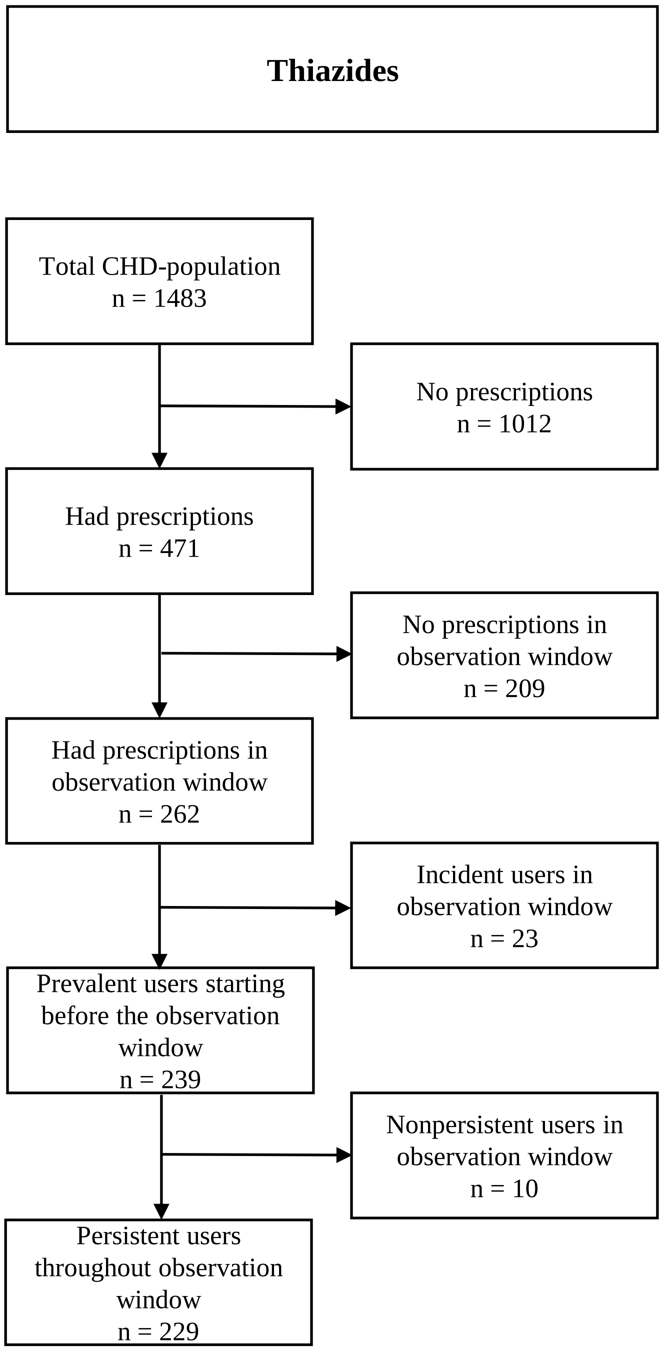
Thiazides
Total CHD-population n = 1483
No prescriptions n = 1012
Had prescriptions n = 471
No prescriptions in observation window n = 209
Had prescriptions in observation window n = 262
Incident users in observation window n = 23
Prevalent users starting before the observation window n = 239
Nonpersistent users in observation window n = 10
Persistent users throughout observation window n = 229
