# Supplementary material for: Medication adherence among persons with coronary heart disease and associations with blood pressure and low-density-lipoprotein-cholesterol
Source: Eur J Clin Pharmacol. 2022 Jan 21;78(5):857–67. doi: 10.1007/s00228-022-03276-4 (PMC9005431; doi:10.1007/s00228-022-03276-4)
Supplement: Supplementary file 3 — Supplementary file3 (PDF 203 KB) [file 228_2022_3276_MOESM3_ESM.pdf]

Supplementary 3: Lifestyle variables

Explanation for making and interpreting the lifestyle variables:

We constructed two variables containing a gradient of lifestyle including ordinal variables on alcohol consumption, smoking, diet and physical activity (see table below).

The variables were made in R using the package “vegan” (see R code below). First the dissimilarity in the original variables were calculated using gower distance which is the preferred choice for heterogeneous variables. This distance matrix was then used to identify four clusters using hierarchical clustering. Next, multidimensional scaling was preformed on the distance matrix made earlier, giving the sets of points with distances equaling the dissimilarities in the different variables used (alcohol consumption, smoking, diet and physical activity, see table below). The points from the multidimensional scaling were saved as the two lifestyle variables used in further analyses.

The points from the multidimensional scaling with colors indicating the clusters identified are shown in the figure below. To show the correlation with age, red isolines of age is added onto the plot. To see which of the original variables contribute to the dissimilarities in lifestyle we conducted a correspondence analysis as shown in the second plot in the figure. This shows that lifestyle variable 1 is influenced by especially alcohol consumption, but also consumption of read meat and smoking, so the participants represented by the blue and red colored clusters have a higher consumption of alcohol and red meat and are more likely to be smokers, while the opposite is shown for those in the green and orange clusters. Lifestyle variable 2 appears to be more influenced by intake of cod liver oil or omega 3, where those in the green and orange clusters more often take cod liver oil or omega 3.

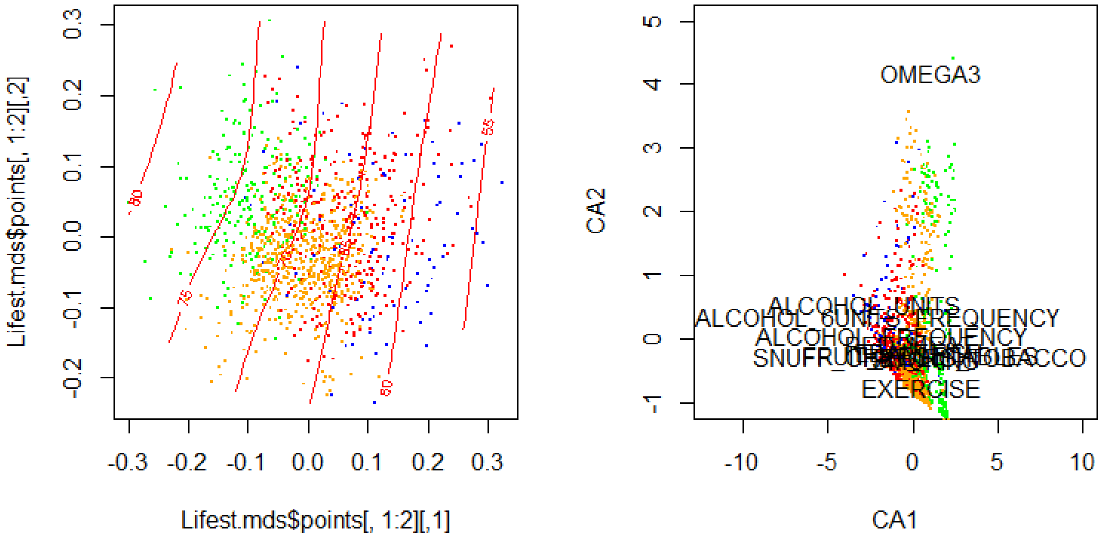

Figure: Plots of the first two dimensions of the multidimensional scaling analyses (left) and the correspondence analysis (right), both with colors indicating the clusters identified

Table: Distribution participants' answer to variables included in new latent lifestyle variables

	Study population n = 1483		Users of lipid-lowering drugs n = 1003		Users of antihypertensive drugs n = 1046		Users of acetylsalicylic acid n = 1042	
	n	(%)	n	(%)	n	(%)	n	(%)
Alcohol frequency								
Never	157	(10.6)	110	(11.0)	124	(11.9)	111	(10.7)
Monthly or less frequently	297	(20.0)	184	(18.3)	216	(20.7)	198	(19.0)
2-4 times a month	593	(40.0)	409	(40.8)	396	(37.9)	424	(40.7)
2-3 times a week	313	(21.1)	214	(21.3)	220	(21.0)	221	(21.2)
4 or more times a week	122	(8.2)	85	(8.5)	89	(8.5)	87	(8.3)
Alcohol units usually drunk when drinking alcohol								
0	157	(10.6)	109	(10.9)	125	(12.0)	110	(10.6)
1-2	717	(48.4)	481	(48.0)	520	(49.7)	499	(47.9)
3-4	472	(31.8)	323	(32.2)	309	(29.5)	339	(32.5)
5 or more	131	(8.8)	86	(8.6)	86	(8.2)	89	(8.5)
Alcohol more than 6 units frequency								
Never	852	(57.5)	585	(58.3)	643	(61.5)	606	(58.2)
Less frequently than monthly	478	(32.2)	313	(31.2)	296	(28.3)	334	(32.1)
Monthly	101	(6.8)	68	(6.8)	69	(6.6)	69	(6.6)
Weekly	27	(1.8)	18	(1.8)	17	(1.6)	15	(1.4)
Daily or almost daily	5	(0.3)	3	(0.3)	5	(0.5)	4	(0.4)
Smoking								
Now, daily	196	(13.2)	122	(12.2)	124	(11.9)	135	(13.0)
Now, sometimes	31	(2.1)	19	(1.9)	19	(1.8)	19	(1.8)
Previously	883	(59.5)	639	(63.7)	645	(61.7)	650	(62.4)
Never	373	(25.2)	223	(22.2)	258	(24.7)	238	(22.8)
Snuff or chewing tobacco								
Now, daily	91	(6.1)	62	(6.2)	61	(5.8)	63	(6.0)

Now, sometimes	2	(0.1)	2	(0.2)	2	(0.2)	2	(0.2)
Previously	76	(5.1)	45	(4.5)	40	(3.8)	50	(4.8)
Never	1311	(88.4)	892	(88.9)	940	(89.9)	924	(88.7)
Red meat								
0-1 times per month	88	(5.9)	57	(5.7)	58	(5.5)	61	(5.9)
2-3 times per month	322	(21.7)	224	(22.3)	222	(21.2)	221	(21.2)
1-3 times per week	926	(62.4)	621	(61.9)	667	(63.8)	663	(63.6)
4-6 times per week	91	(6.1)	59	(5.9)	53	(5.1)	58	(5.6)
Once a day or more	14	(0.9)	10	(1.0)	7	(0.7)	10	(1.0)
Fruits/vegetable/berries								
0-1 times per month	15	(1.0)	11	(1.1)	11	(1.1)	12	(1.2)
2-3 times per month	56	(3.8)	33	(3.3)	37	(3.5)	38	(3.6)
1-3 times per week	314	(21.2)	221	(22.0)	215	(20.6)	227	(21.8)
4-6 times per week	413	(27.9)	282	(28.1)	295	(28.2)	286	(27.4)
Once a day or more	654	(44.1)	432	(43.1)	461	(44.1)	458	(44.0)
Lean fish								
0-1 times per month	49	(3.3)	25	(2.5)	22	(2.1)	22	(2.1)
2-3 times per month	201	(13.6)	134	(13.4)	124	(11.9)	134	(12.9)
1-3 times per week	1020	(68.8)	703	(70.1)	738	(70.6)	740	(71.0)
4-6 times per week	167	(11.3)	109	(10.9)	125	(12.0)	119	(11.4)
Once a day or more	14	(0.9)	11	(1.1)	10	(1.0)	8	(0.8)
Fat fish								
0-1 times per month	163	(11.0)	103	(10.3)	114	(10.9)	101	(9.7)
2-3 times per month	502	(33.9)	340	(33.9)	351	(33.6)	356	(34.2)
1-3 times per week	696	(46.9)	477	(47.6)	494	(47.2)	501	(48.1)
4-6 times per week	64	(4.3)	42	(4.2)	48	(4.6)	46	(4.4)
Once a day or more	21	(1.4)	15	(1.5)	11	(1.1)	15	(1.4)
Omega 3 or cod liver oil								
Never	1067	(71.9)	717	(71.5)	758	(72.5)	740	(71.0)
Sometimes	184	(12.4)	123	(12.3)	131	(12.5)	136	(13.1)
Daily during the winter season	57	(3.8)	34	(3.4)	33	(3.2)	36	(3.5)

Daily	137	(9.2)	103	(10.3)	97	(9.3)	107	(10.3)
Exercise frequency								
Never	143	(9.6)	88	(8.8)	114	(10.9)	98	(9.4)
Less than once a week	194	(13.1)	126	(12.6)	138	(13.2)	128	(12.3)
Once a week	202	(13.6)	141	(14.1)	153	(14.6)	145	(13.9)
2-3 times a week	541	(36.5)	372	(37.1)	366	(35.0)	381	(36.6)
Approximately every day	369	(24.9)	255	(25.4)	253	(24.2)	269	(25.8)

R-code:

```
#Loading relevant libraries
library(foreign)
library(mgcv)
library(vegan)

#Including relevant variables
Lifest<-pred_ext[,c(7, 31:36, 38:41, 43)]
colnames(Lifest) <- c('AGE', 'ALCOHOL_FREQUENCY', 'ALCOHOL_UNITS',
'ALCOHOL_6UNITS_FREQUENCY', 'SMOKING', 'SNUFF_CHEWING_TOBACCO',
'OMEGA3', 'RED_MEAT', 'FRUIT_VEGETABLES', 'LEAN_FISH', 'FAT_FISH',
'EXERCISE')

#Making Gower distance matrix and defining clusters
Lifest.D<-vegdist(na.omit(Lifest[,-1]),method="gower")
Lifest.hclust<-hclust(Lifest.D)
plot(Lifest.hclust)
Lifecl4<-cutree(Lifest.hclust,4)


#Making and plotting results from multidimensional scaling
par(mfrow=c(1,2))
Lifest.mds<-cmdscale(Lifest.D, eig=T)
plot(Lifest.mds$points[,1:2],type="n")
points(Lifest.mds$points[,1:2],pch=".",cex=2,col=c("red","orange","green","blue")[Lifecl4])
ages<-Lifest[as.numeric(rownames(na.omit(Lifest[,-1]))),1]
ordisurf(Lifest.mds,ages,add=T)

#Running and plotting correspondence analysis to determine which variables contribute to the
#lifestyle variables
Lifest.ca<-cca(na.omit(Lifest[,-1]))
plot(Lifest.ca$CA$u,type="n", xlim = c(-5.5,10), ylim = c(-1,3))
points(Lifest.ca$CA$u,pch=".",cex=2,col=c("red","orange","green","blue")[Lifecl4])
text(Lifest.ca$CA$v,labels=rownames(Lifest.ca$CA$v))
```
